# Time to recovery from malnutrition and its predictors among human immunodeficiency virus positive children treated with ready‐to‐use therapeutic food in low resource setting area: A retrospective follow‐up study

**DOI:** 10.1002/hsr2.959

**Published:** 2022-11-29

**Authors:** Martha kassahun Zegeye, Aysheshim kassahun Belew, Addisalem Damtie Aserese, Derese Bekele Daba

**Affiliations:** ^1^ Department of Public Health College of Medicine and Health Science, Ambo University Ambo Ethiopia; ^2^ Department of Human Nutrition Institute of Public Health, University of Gondar Gondar Ethiopia; ^3^ Department of Public Health College of Medicine and Health Science, Arbaminch University Arbamich Ethiopia

**Keywords:** Ethiopia, malnutrition, ready to use therapeutic food, time to recovery, treatment outcome

## Abstract

**Background and Aim:**

Malnutrition is a serious public health issue and a frequent impact of human immunodeficiency virus (HIV) infection, which raises the risk of morbidity and mortality in affected people. Despite the World Health Organization's (WHO) support for the use of ready‐to‐use therapeutic foods (RUTF) to treat malnutrition, research on the length of time it takes for children with HIV infection to recover from malnutrition and the factors that predict it is lacking, particularly Ethiopia.

**Methods:**

An institution‐based retrospective follow‐up study was carried out in the Amhara regional state referral hospitals in Northern Ethiopia. From 2013 to 2018, a total of 478 children who received RUTF treatments were chosen using a simple random sampling technique. To calculate the likelihood of recovery and the median recovery period, incidence and Kaplan–Meier survival analyses were performed. The Cox regression model was used to identify predictors of time to recovery from malnutrition. The multivariable model only included variables with a *p* value below 0.2. While factors were deemed to be substantially linked with the outcome variable if their *p* value was less than 0.05.

**Results:**

The median recovery duration was 5 months (95% confidence interval [CI] = 4–5 months), and the nutritional recovery rate was 64.64% (95% CI = 60.2–68.9). Moderate acute malnutrition (adjusted hazard ratio [AHR] = 4.60, 95% [CI] = 2.85–7.43), WHO clinical stage I (AHR = 4.01, 95% CI = 1.37–11.77), absence of opportunistic infection (AHR = 1.76, 95% CI = 1.19–2.61), haemoglobin (Hgb) count above the threshold (AHR = 1.36, 95% CI = 1.01–1.85) and family size of 1–3 (AHR = 2.38, 95% CI = 2.38–5.00) were significantly linked to rapid recovery from malnutrition.

**Conclusion:**

In comparison to the period specified by the national guideline (3 months for moderate and 6 months for severe acute malnutrition), the median time to recovery was lengthy. Acute malnutrition, clinical stage, opportunistic infection, Hgb count, and family size were statistically associated with early recovery from malnutrition.

## 1 INTRODUCTION

Malnutrition is defined by the World Health Organization (WHO) as deficiencies, excesses, or imbalances in a person's consumption of nutrients and/or energy.^1^ Stunting (low Height‐for‐Age), wasting (low Weight‐for‐Height [WFH]), and underweight (low Weight‐for‐Age) are the most prevalent types of malnutrition brought on by energy deficits.^2^ Children's malnutrition is a significant public health issue in countries with low resources, such as Ethiopia.^3^ It is also a crucial contributor to the progression of the disease and one of the main side effects of human immunodeficiency virus (HIV) infection and acquired immunodeficiency syndrome (AIDS).^4^

The poor nutritional status of children has a negative effect on HIV‐infected patients through compromised food intake, alterations in intermediary metabolism, and nutrient mal‐absorption.^4^ Furthermore, it decreases patients' levels of micronutrients necessary for the proper operation of acquired and innate immunity, which raises the risk of morbidity and mortality, increases the severity of disease, and delays recovery in HIV‐infected children.^5^

Malnutrition's impact on HIV patients is primarily observed in low‐ and middle‐income nations.^6^, ^7^ According to a study conducted in Africa, malnutrition affects children with HIV at a rate of 42%.^8^ Similarly, 60.2% of HIV‐positive children in a research conducted in west Gojjam, Ethiopia, experienced malnutrition.^9^

The nutritional condition of children with HIV infection is influenced by a variety of factors, including the child's sex,^10^, ^11^ age,^12^ concomitant diseases such tuberculosis, oral ulcer, diarrhea,^13^ and history of hospital admission.^14^

To lessen the impact of malnutrition and enhance nutrition delivery services, Ethiopia has developed the National Nutrition Program. As part of an Outpatient Therapeutic Program employing ready‐to‐use therapeutic food (RUTF), a community‐based preventative food and nutrition intervention is one method for enhancing the nutritional status of HIV/AIDS patients).^15^

In the past, children with severe wasting, underweight, or both severe wasting and underweight received RUTF. Due to increased donor support, the clinical nutrition treatment program now accepts individuals with HIV and children with moderate acute malnutrition (MAM).^16^ Children with MAM and severe acute malnutrition (SAM) who have a strong appetite and no medical issues are managed with RUTF.^12^ Despite the availability of this medication, there are still many children with HIV who are malnourished. Additionally, the efficiency of RUTF and anti‐retroviral treatment (ART) therapies and their results have been studied in very few studies in Low and Middle‐Income Countries. However, the studies looking at the start of ART among patients receiving RUTF concentrated on wasting state alone, and the relationship between the length of RUTF treatment and nutritional status has not yet been examined. Therefore, the purpose of this study is to evaluate how long it takes for HIV‐positive children who received treatment in a resource‐limited context in Ethiopia to recover from malnutrition and the factors that influence it.

## 2 MATERIALS AND METHODS

### 2.1 Study design and setting

A retrospective follow‐up study was carried out at ART clinics and referral hospitals in the Amhara regional states to determine the length of time it took for HIV‐positive children enrolled in the RUTF treatment program from September 30, 2013, as well as the factors that predicted when they would recover from malnutrition. The Amhara regional state is located in Ethiopia's northern part. Five referral hospitals offer chronic HIV care (ART) services together with other services in the Amhara regional state. Around 2975 children had received ART follow‐up in these hospitals at the time of data abstraction. The study was carried out in the four referral hospitals that were chosen.

### 2.2 Sample size determination and sampling procedure

The sample size was determined by taking into account predictors from a prior study that were strongly related to the length of time it took for malnutrition to recover after RUTF treatment^17^ using either STATA or Schoenfeld formula.^18^ Schoenfeld formula is written as follows: $E=\frac{{\left(\frac{Z\alpha }{2}+Z\beta \right)}^{2\;}}{p1p2{\left(\ln\mathrm{HR}\right)}^{2\;}}$ and $n=\frac{E}{P\left(E\right)}$, where *E* = number of required event, *n* = sample size, HR is hazard ratio of selected significant covariates, *p*1 is the proportion of subjects under the exposure group (Marasmus), *p*2 is the proportion of subjects under the nonexposed group (kwashiorkor) p2=1−p1 and P(E) is the probability of an event from the previous study.

Assuming, the Probability of type I error or alpha as 0.05, Power of the study as 80%, and withdrawal probability of 0.075 which is the proportion of subjects who defaulted from a study conducted in Sidama zone, Southern Ethiopia, and by taking the hazard ratio (HR) of 1.53 form weight at admission.

Assuming that the likelihood of type I error, or alpha, was 0.05, the study's power was 80%, the withdrawal probability as 0.075 (i.e., the percentage of individuals that dropped out of the study), and HR of 1.53 form weight at admission. To account for any potential losses during multistage sampling, the final sample size of 239 was multiplied by two, resulting in a total sample size of 478.

From the four referral hospitals, 805 patient cards were found to be eligible. More specifically, in Gondar comprehensive and specialized referral hospital 233, Felege Hiwot referral hospital 265, Debre Markos referral hospital 154, and Debre Birhan referral hospital 153 patient cards were eligible. After the proportional allocation of the sample size, the study subjects were selected by simple random sampling (see Supporting Information Material).

### 2.3 Eligibility criteria

Complete records of malnourished HIV‐positive children aged between 18 months to 18 years who had been under RUTF treatment from 2013 to 2018. The study did not include any records of teenage pregnancies due to the need for additional nutritional concerns when an HIV‐positive woman becomes pregnant.

### 2.4 Data collection methods and procedures

The patient follow‐up cards, RUTF registration books, computerized information databases, and patient follow‐up cards were all employed as data sources. Additional clinical records were gathered, including test results for biomarkers and any pertinent investigations. The card number from the health management information system was utilized to identify specific patient cards. Patient cards were used to capture socio‐demographic information, baseline, follow‐up clinical data, and laboratory information. Data was collected from the start of RUTF to the end of follow‐up time.

### 2.5 Operational definition

#### 2.5.1 Censored

Those who were not experiencing recovery from undernutrition until the end of the study, died before experiencing recovery within the study period, and lost follow‐up before experiencing recovery within the study period by reason not related to the event were counted as censored.^19^

#### 2.5.2 Children

A child is a person 18 years or younger unless national law defines a person to be an adult at an earlier age.^14^

#### 2.5.3 The event of interest

The event of interest is recovery from malnutrition during the follow‐up period.

#### 2.5.4 Recovery

A participant who had been declared as recovered from the RURF registration book by the criteria middle upper arm circumference ≥12 cm for children between 18 months to 5 years and children older than 5 years WFH median percentile >80% for two consecutive visits within 3 or 6 months for MAM and SAM, respectively.^12^

At‐risk children are children who had been diagnosed as having under‐nutrition kept under treatment and are expected to recover from malnutrition under the influence of therapy.

### 2.6 Data processing and analysis

Data were taken from the RUTF registration book and individual medical records, imported into Epi‐Info 7, and then analyzed using STATA version 14 for Windows.

The median (interquartile range [IQR]) for continuous data and the frequency distribution for categorical data were used to describe the patient follow‐up characteristics. Categorical variables were subjected to Chi‐square (*χ* ^2^) tests. The cumulative proportion of recovery among malnourished children at various times was estimated using Kaplan–Meier table analysis. After the start of the RUTF treatment, nutritional recovery time was estimated using Kaplan–Meier, and the log‐rank test was performed to compare nutritional recovery times between groups. The random effect was checked using shared frailty and found to be insignificant. The proportional hazard assumption was verified by looking at Log (‐Log) S (t) plots and the Schoenfeld residual global test before the COX bivariate proportional regression model was used to evaluate the association between nutritional recovery time and variables. A multivariate COX‐proportional adjusted model was used to find independent variables of nutritional recovery time. To proclaim the existence of a significant association between nutritional recovery time and variables, *p* values less than 0.05 were utilized to estimate the adjusted HRs with their 95% confidence intervals (CI).

### 2.7 Ethical clearance and consent to participate

The Institutional Review Board of the University of Gondar (IRB‐UoG) examined the study's detailed methodology and gave its approval before obtaining ethical clearance. A formal letter of support was provided to the administrative staff of the respective referral hospitals (Gondar comprehensive and specialized referral hospital, Felege Hiwot referral hospital, Debre Markos referral hospital, and Debre Birhan referral hospital) then permission was secured to access patient cards, registration books, Health management information system database and others. Confidentiality of extracted data and information was ensured at all levels obeying to Declaration of Helsinki.

## 3 RESULTS

### 3.1 Socio‐demographic characteristics of study participants

A total of 478 patient cards were reviewed in this study of which 57.74% were males. About, 35.77% of the study participants were in the age group of 10–14 years with a median age of 11 years IQR (7, 11) years. Nearly 77% of the respondents were urban residents and the majority (80.17%) of the study participants lived with their families. About family history, 52.72% of the children had been living in a family size of 4–6 family members. Regarding the age and marital status of the caregiver, 44.56% and 48.54% of caregivers were in the age range of 30–40 years and were married, respectively whereas 28.03% of caregivers were housewives and 39.75% were illiterates (Table 1).

**Table 1:** Socio‐demographic characteristics of malnourished HIV‐positive children and their caregivers, who were under RUTF in Amhara regional state referral hospitals, from 2013 to 2018

Variable	Frequency (*n*)	Percent (%)
Sex		
Male	276	57.74
Female	202	42.26
Age		
<5	43	9.00
5–9	151	31.59
10–14	171	35.77
15–18	113	23.64
Residence		
Urban	370	77.41
Rural	108	22.59
Parental status		
Both alive	264	55.23
Mother or father alive	156	32.64
Both dead	58	12.13
Age of the caregiver		
≤29	84	17.57
30–39	213	44.56
40–49	129	26.99
≥50	52	10.88
Relation to the child		
Parent	385	80.54
Sister/brother	27	5.65
Uncle/aunt	23	4.81
Grandparent	25	5.23
Other	18	3.77
Religion		
Orthodox	420	87.86
Muslim	35	7.32
Other	23	4.82
Marital status		
Single	50	10.64
Married	232	48.54
Divorced	43	9.00
Widowed	153	32.01
Educational status		
Illiterate	190	39.75
Primary school	172	35.98
Secondary school	57	11.92
College/university	59	12.34
Occupation		
Government employee	66	13.81
Nongovernment employee	63	13.18
Housewife	134	28.03
Daily laborer	78	16.32
Self‐employed	103	21.55
Farmer	21	4.39
Other	13	2.72
Family size		
1–3	183	38.28
4–6	252	52.72
≥7	43	9.00

Abbreviations: HIV, human immunodeficiency virus; RUTF, ready‐to‐use therapeutic foods.

### 3.2 Physical examination, ART/HIV status, laboratory, results, and medications

Regarding the medical history of the study participants, 76.15% of the participants had MAM at the time of admission. The majority (85.77%), of the children were on ART of which 53.77% of the participants had not disclosed their HIV status at the start of RUTF. Furthermore, around 64.44% of them were in WHO clinical stage I. This study also indicated that 64.01% of the participants had a CD4 count of 500 cells/μl and above while 56.90% and 25.73% of the patient had taken Cortimoxazole and isoniazid (INH) prophylaxis, respectively. In addition to the above medical history, records indicated that 70.29% of the study participants had age‐appropriate vaccination despite developing opportunistic infections (42.05%) of the study participants (Table 2).

**Table 2:** Type of malnutrition, HIV/ART status, laboratory results, and medication profile of HIV‐positive malnourished children who were under RUTF in Amhara regional state referral hospitals, Ethiopia, 2013 to 2018

Variables	Frequency (*n*)	Censored number (%)	Recovered number (%)
Type of malnutrition			
MAM	364	103 (28.3)	261 (71.7)
SAM	114	66 (57.90)	48 (42.10)
Disclosure			
Not disclosed	257	84 (32.68)	173 (67.32)
Disclosed	203	76 (37.44)	127 (62.56)
Partially disclosed	18	9 (50)	9 (50)
ART status			
On ART	410	141 (34.39)	269 (65.60)
Eligible but not on ART	35	19 (54.29)	16 (45.71)
Pre ART	33	9 (27.27)	24 (72.73)
ART adherence			
Good	343	92 (26.82)	251 (73.18)
Fair and poor	72	49 (68.1)	23 (31.9)
WHO clinical stage			
Stage I	308	63 (20.45)	245 (79.55)
Stage II	133	75 (56.39)	58 (43.61)
Stage III & IV	37	31 (83.78)	6 (16.22)
Hgb threshold			
Below	241	119 (49.17)	123 (50.83)
Above	237	50 (21.19)	186 (78.81)
CD4 count			
≥500 cells/μl	306	82 (26.80)	224 (73.20)
200–499 cells/μl	132	61 (46.21)	71 (53.79)
<200 cells/μl	40	26 (65)	14 (35)
Cotrimoxazole			
Yes	272	104 (38.24)	168 (61.76)
No	206	65 (31.55)	141 (68.45)
INH prophylaxis			
Yes	123	60 (48.78)	63 (51.22)
No	355	109 (30.70)	246 (69.3)
Vaccination status			
Appropriate for age	336	99 (29.46)	237 (70.54)
Not appropriate for the age	23	10 (43.48)	13 (56.52)
Not vaccinated	83	45 (54.22)	38 (45.78)
Unknown	36	15 (41.67)	21 (58.33)

Abbreviations: ART, antiretroviral therapy; Hgb, haemoglobin; HIV, human immunodeficiency virus; INH, isoniazid; MAM, moderate acute malnutrition; RUTF, ready‐to‐use therapeutic foods; SAM, severe acute malnutrition; WHO, World Health Organization.

### 3.3 Incidence of recovery

**Figure 1:**
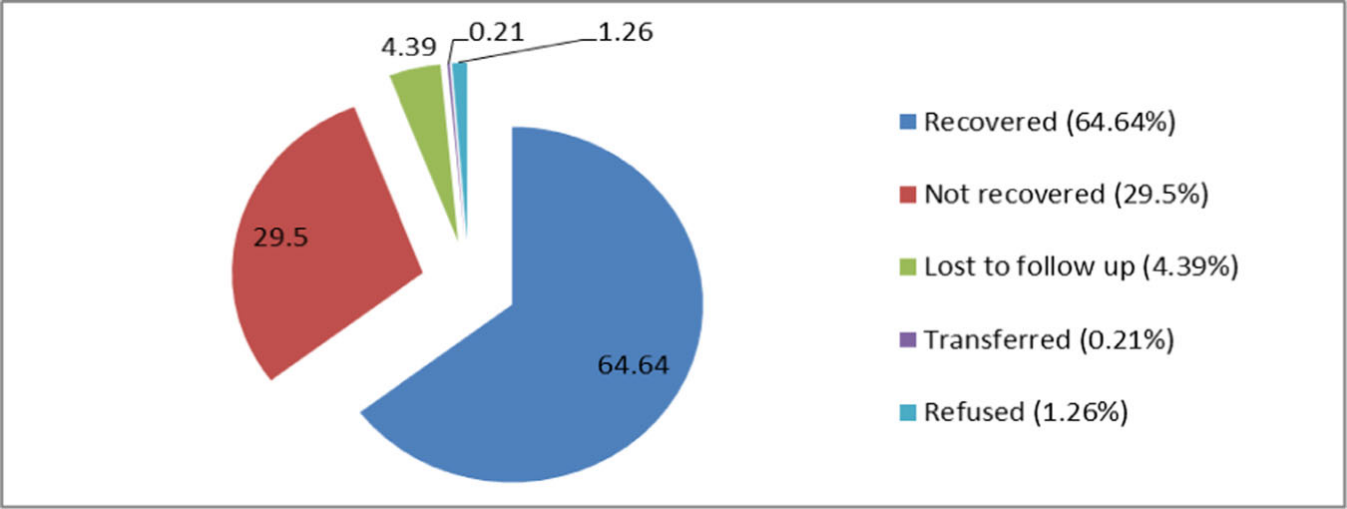
Final status of malnourished HIV‐positive children followed up in Amhara regional state referral hospitals, Ethiopia from 2013 to 2018 (*n* = 478). HIV, human immunodeficiency virus.

The children were monitored for a minimum of 2 months and a maximum of 6 months, with a mean follow‐up period of 4 months (*SD* ± 1 month). During follow‐up, a total of 309 (64.64%) children recovered from malnutrition (Figure 1).

The incidence density was around 15 cases per 100 person‐months. The total person‐time was 2058 months. The incidence rate of nutritional recovery in children with MAM patients was 18 children per 100 person‐months while for SAM patients it was 8 children per 100 person‐months.

### 3.4 Time to recovery from malnutrition after the initiation of RUTF

**Figure 2:**
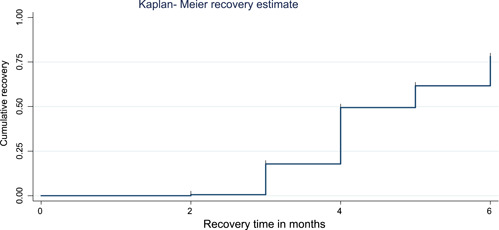
Kaplan–Meier graph of malnourished HIV‐positive children followed‐up among referral hospitals in Amhara regional state, Ethiopia, from 2013 to 2018. HIV, human immunodeficiency virus.

In our study, the median recovery time from malnutrition was 5 months (95% CI = 4–5). The cumulative proportion of recovery was 0.6% at 2 months, 17.5% at 3 months, 49.5% and 78% at 5 and 6 months, respectively (Figure 2).

### 3.5 Factors predicting recovery

To identify statistically significant predictors of recovery from malnutrition among HIV‐positive children in Amhara regional state referral hospitals in Ethiopia, candidate variables were first screened using a univariate Cox regression analysis. Variables with a p‐value of less than 0.2 were taken into consideration as candidates and entered into a multivariable Cox regression model.

After adjustment for other variables, the time it takes to recover from malnutrition was 4.6 times earlier for children with MAM than compared to children with SAM [adjusted hazard ratio (AHR) = 4.60 (95% CI = 2.85–7.43)]. Regarding the presence of opportunistic infections, those without opportunistic infections were 76% more likely to recover faster than those with opportunistic infections [AHR = 1.76 (95% CI = 1.19–2.61)]. Similarly, those with hemoglobin counts above the threshold were 36% more likely to recover faster when compared to children with a hemoglobin count below the threshold [AHR = 1.36 (95% CI = 1.01–1.85)]. Being at WHO clinical stage I and living in a family with a family size of 1–3 members, were more likely to recover earlier by 4 times [AHR = 4.01 (95% CI = 1.37–11.77)] and 2.4 times [AHR = 2.38 (95% CI = 1.14–5.00)], respectively (Table 3).

**Table 3:** Cox regression analysis for predictors of time to recovery from malnutrition among HIV‐positive children in Amhara regional state referral hospitals, Ethiopia from 2013 to 2018, (*N* = 478)

Variables	No. at risk	Recovered	CHR (95% CI)	AHR (95% CI)
Sex				
Male	276	165	0.84 (0.67–1.05)	1.05 (0.77–1.42)
Female	202	144	1	1
Type of malnutrition				
MAM	364	261	5.14 (3.61–7.32)	4.60 (2.85–7.43)*
SAM	114	48	1	1
Age of the caregiver				
<29	84	57	1	1
30–39	213	146	0.87 (0.64–1.18)	1.01 (0.68–1.52)
40–49	129	72	0.64 (0.45–0.91)	0.87 (0.57–1.40)
>50	52	34	0.60 (0.52–1.23)	1.04 (0.59–1.83)
Marital status				
Single	50	32	1.25 (0.83–1.89)	1.16 (0.65–2.06)
Married	232	163	1.30 (1.00–1.69)	1.22 (0.85–1.75)
Divorced	43	26	1.07 (0.69–1.66)	0.73 (0.40–1.33)
Widowed	153	88	1	1
Educational status				
Illiterate	190	115	1	
Primary school	172	110	1.05 (0.81–1.37)	0.99 (0.70–1.41)
Secondary school	57	40	1.24 (0.85–1.78)	0.96 (0.56–1.64)
Higher education	59	44	1.47 (1.03–2.08)	1.22 (0.53–2.80)
Occupation of the caregiver				
Government employee	66	46	1.14 (0.80–1.62)	0.79 (0.36–1.76)
Nongovernment employee	63	44	0.94 (0.65–1.35)	0.96 (0.60–1.54)
Housewife	134	88	1	1
Daily laborer	78	46	0.74 (0.52–1.06)	1.06 (0.66–1.70)
Self‐employee	103	69	0.99 (0.72–1.35)	1.13 (0.73–1.76)
Farmer	21	11	0.68 (0.36–1.26)	1.40 (0.55–3.59)
Other	13	5	0.51 (0.21–1.28)	0.49 (0.18–1.36)
Family size				
1–3	183	141	4.62 (2.56–8.35)	2.38 (1.14–5.00)*
4–6	252	156	3.03 (1.68–5.47)	1.52 (0.73–3.19)
>6	43	12	1	1
ART adherence				
Good	343	251	3.47 (2.25–5.33)	1.35 (0.75–2.43)
Fair and poor	72	23	1	1
Functional status for children >5 years				
Working	166	122	1.58 (1.23–2.02)	1.14 (0.85–1.54)
Ambulatory	238	131	1	1
Opportunistic infection				
Yes	201	82	1	1
No	277	227	3.27 (1.23–2.02)	1.76 (1.19–2.61)*
Cortimoxazole prophylaxis				
Yes	272	168	0.80 (0.64–1.00)	1.08 (0.81–1.45)
No	206	141	1	1
INH prophylaxis				
Yes	123	63	1	1
No	355	246	1.53 (1.16–2.02)	1.13 (0.79–1.61)
Vaccination				
Appropriate for age	336	237	1.75 (1.24–2.46)	1.12 (0.72–1.73)
Not appropriate for age	23	13	1.40 (0.75–2.63)	1.47 (0.63–3.26)
Not vaccinated	83	38	1	1
Unknown	36	21	1.38 (0.81–2.36)	0.96 (0.50–1.82)
WHO clinical stage				
Stage I	308	245	9.48 (4.20–21.39)	4.01 (1.37–11.77)*
Stage II	133	58	3.32 (1.48–7.69)	2.13 (0.72–6.29)
Stage III & IV	37	6	1	1
Hgb threshold				
Above	237	189	2.32 (1.84–2.93)	1.36 (1.01–1.85)*
Below	241	122	1	1
CD4 count				
≥500	306	224	2.57 (1.50–4.42)	1.28 (0.68–2.40)
200–499	132	71	1.63 (0.92–2.82)	1.15 (0.59–2.21)
≤199	40	14	1	1

Abbreviations: AHR, adjusted hazard ratio; CHR, crude hazard ratio; confidence interval.

* *p* < 0.05.

## 4 DISCUSSION

The recovery rate from malnutrition among HIV‐positive children within 6‐month follow‐up period was below the international minimum standards for recovery from nutritional rehabilitation (>75%).^20^ This might be due to poor adherence to the RUTF treatment protocol. Also, it is lower when compared to the recovery rate found in Zambia 80%.^11^ This disparity is due to the treatment time in the latter case, which added 7 weeks for MAM and 10 weeks for SAM compared to the national standard, while our study was based on treatment outcomes seen within 6 months of follow‐up time. Nonetheless, it was higher than the recovery rate among some other HIV‐positive population studies done in Gondar and sub‐Saharan Africa which showed a recovery rate of 49.5% and 47.4%, respectively.^13^, ^14^ This discrepancy may result from the difference in the magnitude of severely malnourished children. In our study, the majority of the patients were moderately malnourished (76.15%) while it was 62.3% in Gondar and 44.2% in sub‐Saharan Africa.^13^, ^14^ Furthermore, it is high when compared to a study done in Malawi, which showed a recovery rate of 56%.^21^ This digression might be due to the short treatment follow‐up time they used that is 4 months for a study population which includes both MAM and SAM patients. However, the recovery rate of this study was in line with the study done in Mekelle which was 62.2%.^22^

The median recovery period for the entire study population was 5 months (95% CI = 4–5) which is in line with research done in Gondar and Sub‐Sahara Africa (SSA)^13^, ^14^ and relatively shorter when compared to research done in Zambia which was reported as 6 months.^11^ The inconsistency might be due to because the majority of the study participants in this study were MAM patients 76.15% while it was 28.1% in Zambia. Patients with SAM are more likely to have a worse outcome than those with MAM.^23^, ^24^ However, it was relatively long when compared to a study done in southern Ethiopia which showed a median recovery time of 42 days, yet the total follows up time was 2 months and patients were under constant supervision.^17^ Similarly, the mean recovery time seen in research done in Malawi was shorter, which was 86 days.^21^ This discrepancy can be due to the duration of follow‐up time and frequency of check‐ups and RUTF supplementation was based on their body kilogram. However, in our study areas, RUTF supplementation was fixed by the type of malnutrition; that is those children with MAM will receive 90 sachets and 120 sachets for SAM patients. In addition, Patients were also only given counseling at the medical facility at the start of their treatment. This can be as a result of the heavy patient load, which might leave little time for counseling and feedback. Additionally, patients weren't checked in‐between appointments, only at the end of each month. It has been demonstrated that applying stricter follow‐up procedures for children receiving home health care could enhance RUTF results.^24^

Children with moderate malnutrition were 4.5 times more likely to recover earlier than children with SAM. The importance of closely monitoring the nutritional status of HIV patients, treating malnutrition at its earliest stages, and expanding early access to HIV/AIDS care are all emphasized by this result, which is also supported by other community‐based programs for malnutrition carried out in Gondar, Zambia, Malawi, and longitudinal study in SSA.^11^, ^13^, ^14^, ^21^

This study showed that children without opportunistic infections were 1.76 times (95% CI = 1.19–2.61) more likely to recover earlier than those children with opportunistic infections. The presence of infection which results in fever, diarrhea, and vomiting contributes to the failure of RUTF and dalliance of recovery time.^25^, ^26^, ^27^

Moreover, children with WHO clinical stage I were four times more likely to recover earlier than children with advanced WHO clinical stages. It is supported by a similar study conducted in Ethiopia, Sub‐Saharan Africa, and the rural district of Malawi which revealed that WHO clinical stage III and IV at admission as one of the risk factors for failure to respond to RUTF.^13^, ^21^, ^25^

Malnourished children with Hgb levels above the threshold for anemia were 1.36 times (95% CI = 1.01–1.85) more likely to have early nutritional recovery when compared to those children with a Hgb count below the threshold level. This result is supported by research done in Bahirdar (AHR = 1.552, 95% CI = 1.134–2.124).^28^ However, a study that was done in Mekelle and Burkina Faso didn't show a significant association between recovery rate and hemoglobin level.^22^, ^29^ This means that if children are treated in accordance with the protocol for the management of anemia, it does not negatively impact the time needed for nutritional recovery even when they are anaemic at the time of admission. According to this study, the problem could be that children weren't getting iron supplements even if they were anaemic.

Another result found in this study is that a family size greater than 7 members was considered a risk factor for prolonged nutritional recovery time, the main explanation behind could be that, as the family size increases the likelihood of sharing RUTF amongst family member increase as well as other nutritional supplementations and cares given for the child will decrease. This conclusion was reinforced by a qualitative study conducted in Addis Ababa, Ethiopia, which indicated that 1 in 3 patients share food with their children and other HIV‐positive individuals with whom they have comparable health conditions simply because they are unable to avoid doing so due to their culture.^30^

## 5 LIMITATION OF THE STUDY

Certain types of information, such as laboratory results and therapies administered, were lacking for some of the patients since this study is a retrospective follow‐up that is based on a review of routinely gathered data from four referral hospitals. There was no information on the availability of food in the home, dietary intake from other sources, food sharing among family members, or patient compliance for nutrition therapy, which may have tainted the outcomes of our predictive factor analysis.

## 6 CONCLUSION

The finding of this study confirmed that the recovery rate from malnutrition was below the recommended national standard. Level of malnutrition, opportunistic infection, WHO clinical stage, Hgb count at admission, and household family size were associated with recovery time.

## 7 RECOMMENDATION

The federal ministry of Health shall formulate regular and comprehensive nutritional screening programs among children living with HIV/AIDs to improve nutritional treatment outcomes.

Health care professionals shall screen for anemia and initiate treatment as soon as possible for malnourished HIV‐positive children. Additionally, they shall offer early diagnosis and treatment of opportunistic illnesses and advice to caregivers on how to prevent them. Disseminate Health information to caregivers about the need and ways of controlling HIV progression. Finally, family/caregivers of malnourished HIV‐positive children shall work to avoid opportunistic infection, reduce family size and avoid sharing of recommended dietary supplies with other children.

## AUTHOR CONTRIBUTIONS

**Martha kassahun Zegeye**: conceptualization; formal analysis; funding acquisition; investigation; methodology; project administration; software; writing—original draft. **Aysheshim kassahun Belew**: conceptualization; supervision; validation; visualization. **Addisalem Damtie Aserese**: data curation; supervision; visualization. **Derese Bekele Daba**: formal analysis; writing—review & editing.

## CONFLICTS OF INTEREST

The authors declare no conflicts of interest.

## TRANSPARENCY STATEMENT

The lead author Martha kassahun Zegeye affirms that this manuscript is an honest, accurate, and transparent account of the study being reported; that no important aspects of the study have been omitted; and that any discrepancies from the study as planned (and, if relevant, registered) have been explained.

## Supporting information

Supplementary information.

## Data Availability

All data that support the findings of this study are available from the corresponding author upon request.
