# Supplementary material for: Time to recovery from malnutrition and its predictors among human immunodeficiency virus positive children treated with ready‐to‐use therapeutic food in low resource setting area: A retrospective follow‐up study
Source: Health Sci Rep. 2022 Nov 29;6(1):e959. doi: 10.1002/hsr2.959 (PMC9708904; doi:10.1002/hsr2.959)
Supplement: Supplementary file 1 — Supplementary information. [file HSR2-6-e959-s001.docx]

**Amhara regional state referral hospitals**

**(5)**

Gondar

N=311

Felege Hiwot

N=375

Debre Markos

N=215

Debre Birhan

N=204

233

265

154

153

**N=478**

**SRS**

**Screening**

**Complete registries**

139

160

90

89

**SRS**

Fig. Schematic representation of sampling procedure for malnourished HIV positive children followed up among referral hospitals in Amhara regional state, Ethiopia, from 2013 to 2018
